# A Randomized Controlled Trial of a Partially Hydrolyzed Formula on Comfort Measures in Fussy Infants

**DOI:** 10.1016/j.cdnut.2025.107574

**Published:** 2025-10-13

**Authors:** Veronica Fabrizio, Michelle M Bohan Brown, Philip Boucher, Shameza Boyd, Sisi Cao, Christopher Davis, Swati Johnson, Nafees Khan, Nancy Moore, Katosha Muse, Fred Ogwara, Ashley C Patterson, Jennifer L Wampler, Michael Yeiser, Weihong Zhuang, Steven S Wu

**Affiliations:** 1 Evidence Generation and Clinical Research, Mead Johnson Nutrition, Evansville, IN; 2 Indiana University School of Medicine, Evansville, IN; 3 Nutrition Science, Mead Johnson Nutrition, Evansville, IN; 4 Frontier Pediatric Research, Lincoln, NE; 5 Alabama Clinical Therapeutics, Birmingham, AL; 6 Tribe Clinical Research, Greenville, SC; 7 Cedar Health Research, Irving, TX; 8 Mid Valley Research Inc, Moline, IL; 9 AVIATI Healthcare & Clinical Research, Memphis, TN; 10 Springs Pediatrics, Rosharon, TX; 11 Springs Medical Research, Owensboro, KY

**Keywords:** fussiness, crying, spit-up, sleep, polydextrose, galactooligosaccharides

## Abstract

**Background:**

Parent-reported fussiness and crying are common during early infancy and may be alleviated by switching the formula for infants who are formula fed.

**Objectives:**

This study evaluated the nutritive effects of a partially hydrolyzed cow milk protein (PHP) formula with an added prebiotic blend on parent/caregiver-reported fussiness and other measures of comfort in infants with perceived fussiness.

**Methods:**

Infants (15–75 d of age) exclusively receiving a commercial intact protein infant formula and rated moderately, very, or extremely fussy were randomized to receive intact cow milk protein formula (control, *n* = 73) or investigational PHP formula (INV-PHP, *n* = 76). Both formulas had a prebiotic blend of polydextrose and galactooligosaccharides (1:1, 4 g/L). Parent-reported outcomes at baseline (D0), end of study feeding day 1 (D1), and daily over the 28-d study feeding were analyzed using repeated-measures analysis of variance.

**Results:**

Of 149 infants randomly assigned, 127 completed study feeding (control, *n* = 65; INV-PHP, *n* = 62). The primary outcome of parent-reported fussiness was not different between groups. Among secondary outcomes between the groups, there was a significant decrease in crying throughout the study (weeks 1–4; *P* = 0.01) and significantly fewer infants who experienced excessive crying during the study (weeks 1, 2, and 4; *P* ≤ 0.045). Formula intake was not different between groups. Incidences of adverse events were low with no significant group differences. On post hoc analysis, within each group, fussiness, gassiness, and spit-up significantly decreased from D0 to D1 (*P* < 0.001) and was maintained through study end (*P* < 0.001).

**Conclusion:**

In infants with parent-perceived fussiness, a partially hydrolyzed cow milk protein formula with an added prebiotic blend, INV-PHP, does not decrease fussiness when compared with control formula but does reduce crying and excessive crying.

The study was registered at clinicaltrials.gov as NCT05245422.

## Introduction

Fussing and crying in infancy may be associated with stress for infants and caregivers [1]. During infancy, parents often seek pediatrician support to manage fussiness, gassiness, crying, spit-up, and sleep [2]. Excessive crying, sometimes diagnosed as infantile colic using Rome IV criteria [3], occurs in ∼20% of infants and peaks ∼6 wk or ∼42 d of age [4]. Strategies from reassurance to switching formulas, for infants receiving infant formula, have been used to manage fussing and crying [4], including changing from intact cow milk protein formula to partially hydrolyzed cow milk protein (PHP) formula. Although partially hydrolyzed formulas are not indicated for cow milk protein allergy [5,6], they may ease symptoms of digestive discomfort by increasing gastric emptying and decreasing curd formation [7,8]. Previously, decreased crying, spit-up and gas [9], and soft stools [9,10] have been reported in studies of PHP infant formulas.

Prebiotic ingredients are used in infant formula to simulate the functionality of human milk oligosaccharides. The prebiotic blend of polydextrose (PDX) and galactooligosaccharides (GOS; 1:1, 4 g/L) has been demonstrated to promote beneficial stool bacteria, including *Bifidobacteria* [11] and *Lactobacilli* [12]. In healthy term infants, intact protein formula that had the prebiotic blend of PDX:GOS was well-tolerated, supported typical growth, promoted softer stools, accelerated the development of more mature daytime sleep-wake patterns, and shortened parent-reported episodes of crying and fussing [11,[13], [14], [15]] compared with formula with no added PDX:GOS. Previous studies, however, did not include an infant population with perceived fussiness or evaluate added PDX:GOS in PHP formulas.

In the present study, healthy term infants, without underlying disease, with perceived fussiness and who were exclusively formula fed were randomly assigned to receive an investigational (INV) PHP formula (INV-PHP) or intact protein formula (control) over a 28-d feeding period. Both formulas had the PDX:GOS blend. Evaluation of nutritive effects on infant fussiness was the primary objective; digestive discomfort (crying, gassiness, spit-up, and stool patterns), sleep, and parental quality of life were secondary objectives in parent-reported moderately, very, and extremely fussy infants.

## Methods

### Study design and participants

For this multicenter, double-blind, randomized, controlled, parallel-group, prospective trial, mothers who had previously made the decision to exclusively provide infant formula were screened for study eligibility (Table 1). Participants were enrolled (March 2022 to July 2023) at 10 clinical sites in the United States (clinicaltrials.gov: NCT05245422). There was a correction to the clinicaltrials.gov registry at the end of the study to correct a clerical error. The time frame of secondary daily diary outcome measures excluding fussiness where the time frame should be study feeding days 1 to 28 and not days 8 to 28. The participant’s parent(s) or legal guardian provided written informed consent prior to randomization. The research protocol, with statistical analysis plan included, was conducted according to guidelines of the Declaration of Helsinki (including October 1996 amendment). The research protocol, statistical analysis plan, and informed consent forms received approval by a central institutional review board (Advarra Inc) in October 2021 prior to participant enrollment. The study complied with good clinical practices.

**TABLE 1:** Participant inclusion and exclusion criteria at study visit 1 (randomization/D0).

Inclusion criteria	Exclusion criteria
• Primary caregiver has reliable access to the internet and a reliable device (such as a computer, tablet, or smartphone) to access mobile applications and be able to view and complete study questionnaires • Singleton birth • 15–75 d of age at visit 1, inclusive (day of birth is considered day 0) • Gestational age of ≥37–42 wk (36 wk and 6 d is considered 36 wk gestational age) • Birth weight of 2500 g (5 lbs 8 oz) or more • Exclusively receiving an intact protein infant formula (cow milk-based or plant-based) for 7 d prior to visit 1 • Answer to question: “On average, how fussy has your baby been over the past 3 days” is moderately fussy, very fussy, or extremely fussy at visit 1 • Parent(s) or legal guardian has full intention to exclusively feed study formula during the study period • Parent(s) or legal guardian agrees not to enroll infant in another interventional clinical study while participating in this study • Signed informed consent obtained from parent or legal guardian for infant’s participation in the study • Signed authorization obtained from parent or legal guardian to use and/or disclose Protected Health Information for infant from birth through the length of the study period	• Infant has been weighed by an HCP and is identified with inadequate weight gain or failure-to-thrive • Diagnosis or suspicion of cow milk protein allergy by an HCP • Any acute illness within the 3 d prior to visit 1 • Infant has had immunizations or a surgical procedure within the 3 d prior to or on visit 1 • Immunizations are planned for the infant during any of the 7 d after visit 1 • Use of oral, intramuscular, or intravenous antibiotics within the 7 d prior to visit 1 • Infant has had bloody stools (visible to the naked eye) within the 7 d prior to visit 1 • Infant has been taking medication (prescribed and over-the-counter) for gastrointestinal conditions for any of the 7 d prior to visit 1 (however, probiotics are allowed) • Infant has a surgical procedure planned during the study period • A history of underlying metabolic or chronic disease; congenital malformation; or any other condition which, in the opinion of the investigator, is likely to interfere with the ability of the infant to ingest food, the normal growth and development of the infant, or the evaluation of the infant • A history of underlying neurological or organic disease likely to cause fussiness, such as (but not limited to) a doctor’s diagnosis of neonatal abstinence syndrome and inflammatory or orthopedic disorders • Infant is immunocompromised (according to a doctor’s diagnosis of immunodeficiency such as combined immunodeficiencies, DiGeorge syndrome, Wiskott-Aldrich syndrome, severe congenital neutropenia, and secondary immunodeficiencies linked to HIV infection, Down syndrome, or others)

Abbreviation: HCP, health care professional.

### Randomization and study group allocation

The study sponsor created and provided a computer-generated randomization schedule in sealed, opaque, consecutively numbered envelopes for each study site. Study formula was assigned by opening the next sequential envelope from the set at each site. Briefly, eligible infants who were moderately, very, or extremely fussy over the previous 3 d by parent report and fed commercialized intact protein formula over the past 7 days were randomly assigned to receive 1 of 2 study formulas (Mead Johnson Nutrition) (Table 2) [16]: control, intact protein formula (similar to previously marketed Enfamil); or INV-PHP formula.

**TABLE 2:** Nutrient composition per 100 kcal^1^.

Nutrient	Study formula (target values)	
	Control	INV-PHP
Energy (calories per fluid ounce)	20	20
Total protein^2^ (g)	1.9	2.3
Total fat (g)	5.5	5.4
Linoleic acid (mg)	800	800
α-Linolenic acid (mg)	67	71
Arachidonic acid (mg)^3^	29	29
DHA^3^ (mg)	23	23
Total carbohydrate^4^ (g)	11	11
Lactose (% of total carbohydrate)	∼92%	∼50%
Prebiotic blend (g)	0.6	0.6
Vitamin A (IU)	250	250
Vitamin A (μg)	75	75
Vitamin D (IU)	70	70
Vitamin E (IU)	1.69	2.9
Vitamin K (μg)	5	5
Thiamin (μg)	78	102
Riboflavin (μg)	98	105
Vitamin B-6 (μg)	35	102
Vitamin B-12 (μg)	0.15	0.15
Niacin (μg)	550	944
Folate (μg; dietary folate equivalents)	18.3	20
Pantothenic acid (μg)	550	770
Biotin (μg)	3	2
Vitamin C (mg)	10	10
Choline (mg)	33	33
Inositol (mg)	6	6.1
Calcium (mg)	78	100
Phosphorus (mg)	41	67
Magnesium (mg)	8	8
Iron (mg)	0.8	0.8
Zinc (mg)	0.6	0.7
Manganese (μg)	5.9	10
Copper (μg)	70	71
Iodine (μg)	18	15
Selenium (μg)	3.4	3.5
Sodium (mg)	30	39
Potassium (mg)	105	131
Chloride (mg)	75	75

Abbreviations: DHA, docosahexaenoic acid; INV-PHP, investigational partially hydrolyzed cow milk protein formula

1 All nutrients comply with the United States Infant Formula Act [16].

2 Protein sources for control (intact cow milk protein) and for INV-PHP (partially hydrolyzed cow milk protein).

3 Not typically included on label claim panel.

4 Carbohydrate sources for control: lactose and prebiotic blend of polydextrose (PDX) and galactooligosaccharides (GOS; 1:1 ratio; 4 g/L); and for INV-PHP: glucose polymers, lactose, prebiotic blend of PDX and GOS (1:1 ratio; 4 g/L).

The study participants and study site personnel were blinded to the administered formula. The formula cans were coded using a unique, computer-generated product code. Each product code was linked to a distinct label color. Two different color codes were assigned to each formula. This was done to ensure that if the blinding had to be broken for 1 color, the other colors would remain blinded, preserving the integrity of the trial (no colors were unblinded during the trial). Both study formulas were powders and dispensed in 12.5-ounce cans. The color and product code were the only distinguishing features on the cans; the cans were otherwise identical in appearance, including the can type, lid color, font, weight, and preparation instructions. This ensured that the study formulas could not be visually distinguished, maintaining blinding throughout the trial.

Study visit 1 (randomization) corresponded to day 0 (D0; 15–75 d of age); study feeding day 1 began the next morning (D1) and visit 2 (study end; D29+3 d) was scheduled to ensure participants completed 28 study feeding days. In the event of medical emergency, blinding could be broken by study sponsor personnel; however, it was unnecessary to break the study code prematurely.

### Study objectives and outcomes

**FIGURE 1:**
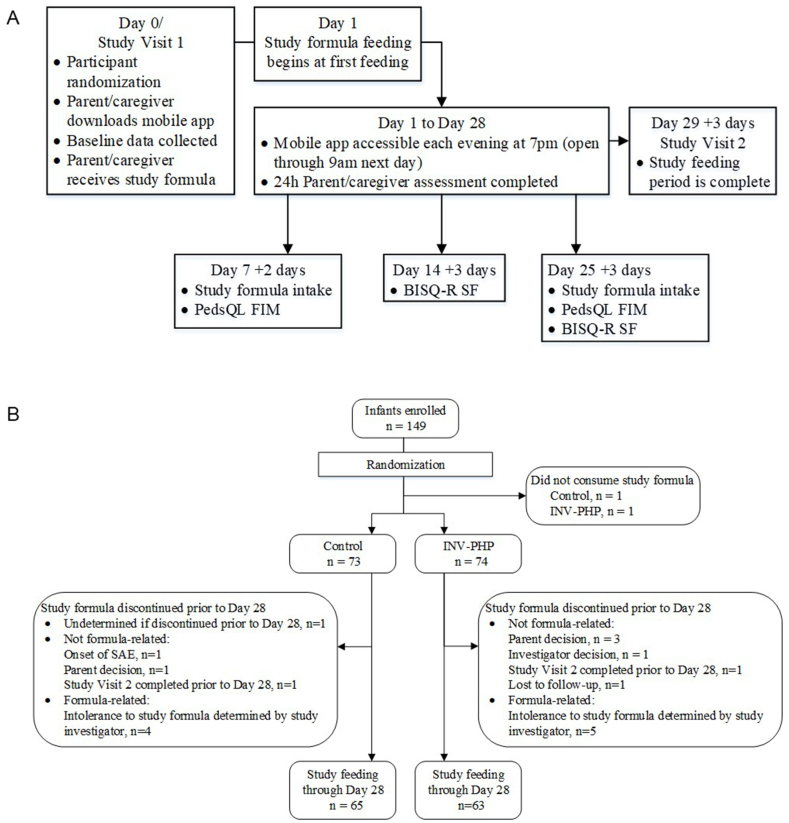
(A) Study timeline and (B) study allocation and participant flow. BISQ-R SF, Brief Infant Sleep Questionnaire-Revised Short Form; INV-PHP, investigational partially hydrolyzed cow milk protein formula; PedsQL FIM, Pediatric Quality of Life Inventory Family Impact Module; SAE, severe adverse event.

The primary outcome was fussiness from D1 to D7. Figure 1A outlines the study event schedule. On D0, the parent/caregiver downloaded a direct data capture mobile application (Laina Enterprises, Inc) and completed a baseline recall for the prior 3-d period: fussiness (time of morning wake-up to 16:00 and 16:00 to bedtime: not fussy = 0; slightly = 1; moderately = 2; very = 3; extremely fussy = 4); amount of gas (none = 1; slight = 2; moderate = 3; excessive = 4); spit-up (number/day); crying (hours/day); stool frequency (number/day); stool consistency (hard = 1; formed = 2; soft = 3; unformed or seedy = 4; watery = 5); night-wakings (number/night); and nighttime sleep quality (very well = 0; well = 1; fairly well = 2; poorly = 3; very poorly = 4). Beginning on D1 through D28, a daily diary became available on the mobile application at 19:00 through 09:00 the next morning to recall outcomes from the preceding 24 h. Week 1 was defined as D1–D7 for all variables except night-wakings and nighttime sleep quality (defined as D2–D8 to reflect previous night’s outcomes but reported as D1–D7 to correspond with study feeding day). Use of nonprescription products for fussiness, gassiness, constipation, spitting up, or pain relief was recorded in the daily diary. Daily diary questionnaire is listed in Supplemental Table 1.

Primary caregivers also completed validated, parent-reported questionnaires via mobile application: Pediatric Quality of Life Inventory Family Impact Module (PedsQL FIM)-Acute (D0, D7, and D25) and Brief Infant Sleep Questionnaire-Revised Short Form (BISQ-R SF) (D0, D14, and D25). The 36-item PedsQL FIM-Acute uses a 5-point scale [17] and is reverse-scored and linearly transformed to a 0–100 scale (0 = 100; 4 = 0; higher scores indicate better functioning). Three composite summary scores are reported based on 6 scales: parent health-related quality of life (HRQoL; physical, emotional, social, and cognitive functioning); family functioning (daily activities and family relationships); and total score [17]. BISQ-R SF evaluates sleep patterns, caregiver perception of sleep, and parent behavior around infant sleep. The design includes 19 scored questions and 1 additional sleep duration question, encompassing 3 subscales: infant sleep, parent perception, and parent behavior. Higher subscale scores (0–100) denote better sleep quality, positive perception of infant sleep, and parent behaviors that promote healthy and independent sleep. Age-based, norm-referenced scoring is based on data from infants and toddlers in the United States [18].

Throughout the study period, immunizations and any use of prescribed medication for gastrointestinal (GI) problems were recorded and the incidence of medically confirmed adverse events (AEs) was collected and coded according to specific event and body system involved.

### Statistical analysis

Perception of infant fussiness score by the primary caregiver during D1 to D7 of study feeding (week 1) was the primary outcome used to compare 2 study groups. Assuming a 0.5-point difference and SD of 1, a sample size of 64 per group was necessary (80% power, α = 0.05, 2-tailed test). Data collected from D0 to D7 for fussiness, gassiness, crying, spit-ups, stool frequency and consistency, night-wakings, and nighttime sleep quality were analyzed using a mixed-model repeated measures (MMRM). For fussiness, the model included study group, day, time period (wake-up to 16:00 and 16:00 to bedtime), all 2-way interactions (study group × day, study group × time period, and day × time period) and the 3-way interaction (study group × day × time period). For all other daily responses, the model included study group, day, and study group × day interaction. To evaluate study feeding through D28, daily responses were averaged for weeks 1–4. Models were similar to those described earlier for the first week of study feeding, except the day term was replaced by week. For crying, 2 outcomes of interest were analyzed: number of hours of crying in 24 h and excessive crying, calculated as crying for ≥3 h/d for ≥3 d/wk, and was analyzed as a binary variable. The number of hours of crying was analyzed using MMRM as described earlier. The proportion of participants with excessive crying was compared between groups using Fisher exact test. Each week was analyzed separately.

BISQ-R SF responses were prepared [19], and subscale scores and total score were analyzed by MMRM. PedsQL FIM total score, parent HRQoL summary score, family functioning summary score, and scale scores were analyzed by MMRM. Terms in the model included study group, time point, and study group × time point interaction. Study formula intake was analyzed by analysis of variance, and medically confirmed AEs were analyzed by Fisher exact test.

All participants who consumed some study formula and had ≥1 parent diary completed after starting study formula were included in the efficacy outcome analyses. Based on previous literature [9], by post hoc analysis, the hypothesis that change from D0 was significantly different from zero was tested for each time point. The predefined and post hoc outcome analyses for all outcome measures are listed in Supplemental Table 2. All *P* values reported are based on 2-tailed tests. All testing was conducted at an α level of 0.05. All analyses were performed using SAS software (version 9.4; SAS Institute).

## Results

### Participants

A total of 149 infants were enrolled and randomly assigned (control, *n* = 74; INV-PHP, *n* = 75); 127 completed 28 d of study feeding (control, *n* = 65; INV-PHP, *n* = 62). Infants who were randomly assigned but were not fed study formula (control, *n* = 1; INV-PHP, *n* = 1) were excluded from analysis (Figure 1B). Group demographics for infant birth characteristics, primary caregivers, and family household were similar (Table 3). No significant group difference in participant age on D1 was detected (range: 12–76 d; mean ± SE—control, 40.4 ± 2.1 d; INV-PHP, 42.5 ± 2.1 d). No group differences in study formula intake (fluid ounce/day) were detected at D7 (control, 27.3 ± 1.6; INV-PHP, 26.5 ± 1.6) or D25 (control, 27.4 ± 1.7; INV-PHP, 28.9 ± 1.7). No group differences were detected in use of prescribed medications for GI problems, immunizations (Table 4), or the number of participants with ≥1 medically confirmed AE (control, *n* = 16, 22%; INV-PHP, *n* = 19, 26%). Incidences of AEs (categorized as: body as a whole; eyes, ears, nose, and throat; GI; metabolic and nutrition; musculoskeletal; respiratory; and skin) were low with no significant group differences. No group differences were detected in overall discontinuation or discontinuation due to study formulas.

**TABLE 3:** Participant and family characteristics at enrollment^1^.

Characteristic	Control	INV-PHP	*P*
Total No. of participants	73	74	
Birth characteristics			
Weight (g)	3324 ± 48.1	3274 ± 47.9	0.468
Sex			0.511
Female	37 (51)	33 (45)	
Male	36 (49)	41 (55)	
Race			0.051
Black	19 (28)	25 (36)	
White	45 (65)	45 (64)	
>1 race	5 (7)	0 (0)	
Ethnicity			1.000
Hispanic	13 (21)	14 (21)	
Not Hispanic	49 (79)	52 (79)	
Baseline fussiness score	1.9 ± 0.1	2.1 ± 0.1	0.287
Age (primary caregiver) at infant’s birth or legal adoption	28.0 ± 0.7	28.5 ± 0.7	0.623
First infant as primary caregiver			0.735
Yes	27 (37)	30 (41)	
No	46 (63)	43 (59)	
No. of people living in the household	4.3 ± 0.2	4.1 ± 0.2	0.470
No. of adult caregivers living in the household	1.9 ± 0.1	2.0 ± 0.1	0.391
No. of children under the age of 18 y living in the household	2.2 ± 0.2	2.2 ± 0.2	0.844
Anyone living in the home smokes			0.518
Yes	11 (15)	16 (20)	
No	62 (85)	59 (80)	
Parent education level			0.823
Partial high school	13 (18)	10 (14)	
High school/general educational development	23 (32)	29 (39)	
Partial college	17 (23)	16 (22)	
Associate degree	8 (11)	1 (1)	
Bachelor degree	5 (7)	12 (16)	
Graduate or professional degree	7 (10)	6 (8)	
Annual household income			0.980
<$25,000	31 (42)	30 (41)	
$25,000–$34,999	11 (15)	7 (10)	
$35,000–$49,999	7 (10)	7 (10)	
$50,000–$74,999	4 (5)	11 (15)	
$75,000–$99,999	4 (5)	1 (1)	
$100,000–$149,999	5 (7)	8 (11)	
$150,000 or more	5 (7)	3 (4)	
Prefer not to answer	6 (8)	6 (8)	

Values are *n* (%) or mean ± standard error.

Abbreviations: INV-PHP, investigational partially hydrolyzed cow milk protein formula.

1 Birth weight, mother’s age at participant’s birth or legal adoption, number of family members living in the household, number of adult caregivers, number of children, and participant days of age on D1 were analyzed by analysis of variance. Annual household income category and parent’s education level were compared by group using the Cochran-Mantel-Haenszel row mean score test. Exposure to smoking in the home (defined as anyone living in the home smoked) and first baby for the primary caregiver were analyzed by Fisher exact test.

**TABLE 4:** Participants (%) by group who had used nonprescription products (to help relieve pain, fussiness, spitting up, constipation, or gassiness), who had immunization, and who had prescribed medications for GI problems during the study period^1^.

Participant who had	Group	*n* (%)		*P*
		Yes	No	
≥1 prescribed medication for GI problems	Control	5 (7)	68 (93)	0.442
	INV-PHP	2 (3)	70 (97)	
≥1 immunization during the first week of study feeding	Control	1 (1)	72 (99)	0.620
	INV-PHP	2 (3)	70 (97)	
≥1 immunization during the study period	Control	15 (21)	58 (79)	0.557
	INV-PHP	18 (25)	54 (75)	
≥1 nonprescription product to help relieve pain, fussiness, spitting up, constipation, or gassiness	Control	12 (17)	60 (83)	0.829
	INV-PHP	14 (19)	58 (81)	

Abbreviations: GI, gastrointestinal; INV-PHP, investigational partially hydrolyzed cow milk protein formula.

1 Compared using Fisher exact test.

### Primary and secondary outcomes between groups

#### Daily diary

**FIGURE 2:**
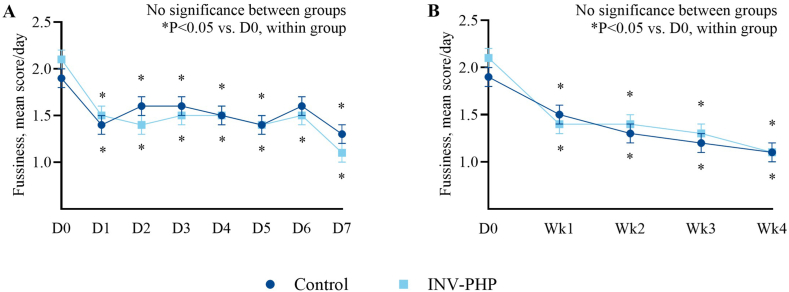
Mean daily values (D1–D7) (A) and weekly averages (week 1 to week 4) (B) compared between groups and within a group to D0 (baseline) for infant fussiness (0–4 scale). Control, dark blue circles; INV-PHP, light blue squares). No between-group differences were detected. ∗*P* < 0.05 for change within group from the baseline value. Bars indicate SEs for means. Sample sizes for each study group at each time point are available in Supplemental Table 3. INV-PHP, investigational partially hydrolyzed cow milk protein formula.

**FIGURE 3:**
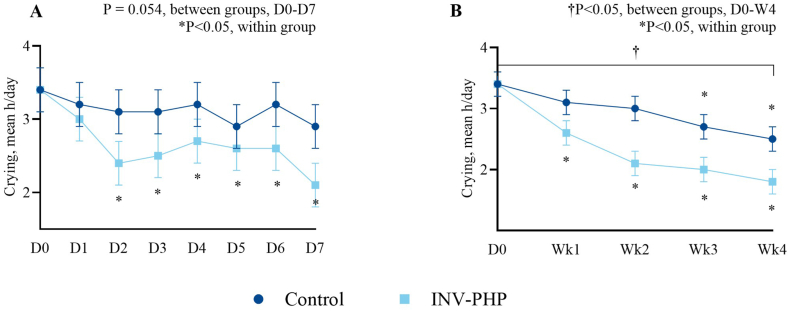
Mean daily values (D1–D7) (A) and weekly averages (week 1 to week 4) (B) compared between groups and within a group to D0 (baseline) for infant crying (0–4 scale). Control, dark blue circles; INV-PHP, light blue squares. ^†^*P* < 0.05 for change between groups; ∗*P* < 0.05 for change within a group from the baseline value. Bars indicate SEs for means. INV-PHP, investigational partially hydrolyzed cow milk protein formula.

**FIGURE 4:**
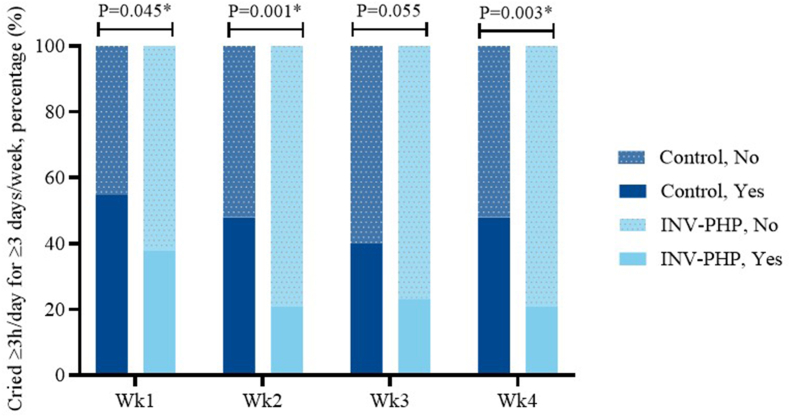
The proportion of infants who cried ≥3 h/d for ≥3 d/wk (represented by dotted compared with plain bar) was compared between groups (control, dark blue; INV-PHP, light blue) during each study week using Fisher exact test; week 1—control, *n* = 71; INV-PHP, *n* = 72; week 2—control, *n* = 66; INV-PHP, *n* = 67; week 3—control, *n* = 62; INV-PHP, *n* = 65; week 4—control, *n* = 64; INV-PHP, *n* = 63. INV-PHP, investigational partially hydrolyzed cow milk protein formula.

No significant difference in mean fussiness (Figure 2; Supplemental Table 3—sample sizes) was found between INV-PHP and control groups. Overall group differences in crying during D0 through D7 were not significantly different (*P* = 0.054); however, significantly less crying was detected for infants in the INV-PHP group compared with those in the control group across the study period (D0 through weeks 1–4; *P* = 0.010) (Figure 3). In addition, the percentage of infants by study group who cried ≥3 h/d for ≥3 d/wk was significantly different (weeks 1, 2, and 4; *P* ≤ 0.045) with fewer participants who cried ≥3 h/d for ≥3 d/wk in the INV-PHP group (Figure 4). In the secondary daily diary outcomes (gassiness, spit-up, crying, stool consistency and frequency, night-wakings, and nighttime sleep quality) (Supplemental Figures 1 and 2), no significant group differences were detected daily (D0–D7) or weekly (D0 to week 1 to week 4) with the exception of crying. Throughout the study period, stool consistency was reported as soft to unformed/seedy in both groups and stool frequency within the mean range of 2 to 3.5 stools/d (Supplemental Figure 2A–D).

### Sleep and quality of life outcomes

No differences in mean BISQ-RF scores by study group were detected at any measured time point (Table 5). For PedsQL FIM-Acute scores, no group differences were detected (Table 6).

**TABLE 5:** BISQ-R SF scores (mean ± SE) by study group at D0, D14, and D25.

BISQ-R SF scale	Time point	Group	*n*	Mean (SE)	*P*, group
Infant Sleep	D0	Control	59	55.1 (2.3)	0.892
		INV-PHP	56	54.2 (2.4)	
	D14	Control	59	62.1 (2.3)∗	
		INV-PHP	57	62.4 (2.3)∗	
	D25	Control	56	61.2 (2.4)∗	
		INV-PHP	57	63.0 (2.3)∗	
Parent behavior	D0	Control	59	81.5 (1.0)	0.854
		INV-PHP	56	82.8 (1.0)	
	D14	Control	59	82.0 (1.0)	
		INV-PHP	57	81.9 (1.0)	
	D25	Control	56	80.9 (1.0)	
		INV-PHP	57	82.1 (1.0)	
Parent perception	D0	Control	59	65.8 (2.3)	0.318
		INV-PHP	56	69.0 (2.3)	
	D14	Control	59	76.2 (2.2)∗	
		INV-PHP	57	77.5 (2.3)∗	
	D25	Control	56	80.1 (2.3)∗	
		INV-PHP	57	81.4 (2.3)∗	
Total score	D0	Control	59	67.4 (1.4)	0.486
		INV-PHP	56	68.7 (1.4)	
	D14	Control	59	73.4 (1.4)∗	
		INV-PHP	57	74.0 (1.4)∗	
	D25	Control	56	74.2 (1.4)∗	
		INV-PHP	57	75.6 (1.4)∗	

Abbreviations: BISQ-R SF, Brief Infant Sleep Questionnaire-Revised Short Form; INV-PHP, investigational partially hydrolyzed cow milk protein formula; SE, standard error.

∗ Compared with D0, *P* ≤ 0.05 within the group.

**TABLE 6:** PedsQL-FIM^1^ scores (mean ± SE) by study group at D0, D7, and D25.

PedsQL-FIM acute scale	Group	Study time point^2^			*P*, group
		D0	D7	D25	
Physical	Control	65.9 (2.2)	69.7 (2.2)	72.0 (2.3)∗	0.835
	INV-PHP	65.3 (2.3)	70.8 (2.3)∗	73.3 (2.4)∗	
Emotional	Control	80.9 (2.3)	82.7 (2.3)	82.1 (2.4)	0.721
	INV-PHP	80.1 (2.3)	81.9 (2.3)	80.6 (2.4)	
Social	Control	81.1 (2.5)	80.5 (2.5)	79.8 (2.6)	0.728
	INV-PHP	79.2 (2.5)	83.4 (2.6)	81.9 (2.6)	
Cognitive	Control	77.7 (2.6)	78.4 (2.6)	78.9 (2.7)	0.934
	INV-PHP	75.4 (2.7)	80.1 (2.7)∗	78.8 (2.8)	
Communication	Control	88.9 (1.9)	88.3 (1.9)	92.0 (2.0)	0.946
	INV-PHP	89.2 (1.9)	89.9 (2.0)	90.7 (2.0)	
Worry	Control	87.2 (2.1)	84.3 (2.2)	86.3 (2.2)	0.955
	INV-PHP	84.7 (2.2)	85.3 (2.2)	88.3 (2.3)	
Daily activities	Control	67.3 (3.0)	69.5 (3.0)	69.0 (3.1)	0.465
	INV-PHP	68.2 (3.0)	71.0 (3.1)	74.5 (3.2)	
Family relationships	Control	84.9 (2.3)	84.8 (2.4)	83.8 (2.4)	0.604
	INV-PHP	84.4 (2.4)	85.7 (2.4)	88.0 (2.5)	
Parent HRQoL summary scale	Control	75.6 (2.0)	77.3 (2.0)	77.8 (2.1)	0.986
	INV-PHP	74.3 (2.0)	78.4 (2.1)∗	78.1 (2.1)	
Family functioning summary score	Control	78.3 (2.3)	79.0 (2.3)	78.2 (2.3)	0.495
	INV-PHP	78.3 (2.3)	80.2 (2.3)	82.9 (2.4)∗	
Total scale score	Control	78.9 (1.8)	79.6 (1.8)	80.2 (1.9)	0.834
	INV-PHP	77.9 (1.8)	80.7 (1.9)∗	81.6 (1.9)∗	

Abbreviations: HRQoL, health-related quality of life; INV-PHP, investigational partially hydrolyzed cow milk protein formula; PedsQL FIM, Pediatric Quality of Life Inventory Family Impact Module.

1 PedsQL FIM-Acute has 36 items using a 5-point scale (0, never a problem; 1, almost never; 2, sometimes; 3, often; and 4, almost always). Responses are reverse scored and linearly transformed to a 0–100 scale (0, 100; 1, 75; 2, 50; 3, 25; and 4, 0; higher scores indicate better functioning).

2 *n* = 67 (D0, control), *n* = 65 (D0, INV-PHP); *n* = 65 (D7, control), *n* = 61 (D7, INV-PHP); *n* = 60 (D25, control), *n* = 56 (D25, INV-PHP).

∗ Compared with D0, *P* < 0.05 within the group.

### Outcome results compared with baseline within group

#### Daily diary

For all outcomes, baseline means (± SE) at D0 were also compared with daily (D1–D7) and weekly (week 1 to week 4) means within study groups by post hoc analysis. In both the control and INV-PHP groups, mean fussiness, gassiness, and spit-up were significantly lower on D1 than those on D0 (*P* < 0.001) and through study end (week 4; *P* < 0.001) (Figure 2A, B—fussiness; Supplemental Table 3—sample sizes for fussiness; Supplemental Figure 1A, B—gassiness; Supplemental Figure 1C, D—spit-up). In the INV-PHP group, significantly less crying was detected by D2 (2.4 ± 0.3 h; *P* < 0.001), which sustained through D7 (2.1 ± 0.3 h; *P* < 0.001) (Figure 3A) and from D0 through week 4 (*P* < 0.001) (Figure 3B). In the control group, no significant differences were detected in crying for D0 compared with that for D1 to D7; however, significantly less crying was detected at weeks 3 and 4 compared with that for D0 (*P* = 0.006 for week 3; *P* < 0.001 for week 4).

Night-wakings significantly decreased from D0 to D1 in both groups (*P* < 0.001) (Supplemental Figure 2E) and continued to be significantly lower through week 4 (*P* < 0.001) (Supplemental Figure 2F). Nighttime sleep quality scores significantly improved from D0 to D1 in both groups (*P* < 0.001) (Supplemental Figure 2G) and continued to be significantly better through week 4 (*P* < 0.001) (Supplemental Figure 2H).

### Sleep and quality of life outcomes

BISQ-RF scores within each group (Table 5) significantly improved from D0 to D14 and D25 for infant sleep (∼5- to 7-point increase), parent perception (∼6 to 10-point increase), and the total score (∼5- to 7-point increase). For PedsQL FIM-Acute scores, statistically significant improvement was detected within group for several subscale scores (Table 6) for D0 compared with those for D7 (INV-PHP: cognitive functioning and parent HRQoL summary scale); D0 compared with those for D25 (control: physical functioning; INV-PHP: family functioning summary score); or D0 compared with those for D7 and D25 (INV-PHP: physical functioning and total scale score).

## Discussion

Infants identified by parent report as moderately, very, or extremely fussy were randomly assigned to receive control (intact protein) or INV-PHP infant formula, both with a prebiotic blend of PDX:GOS, over a 28-d feeding period, and were assessed primarily for fussiness and secondarily for other measures of GI comfort, sleep, and quality of life. The primary outcome of parent-reported fussiness was not significantly different between groups.

Among secondary outcomes, crying and excessive crying decreased significantly in the INV-PHP group than those in the control group. Hours of crying per day were collected in a daily diary, with significantly less crying throughout the study period in infants receiving the INV-PHP than that in those receiving the control formula. Likewise, the percentage of participants who experienced excessive crying was significantly lower in infants receiving the INV-PHP than that in those receiving control formula (weeks 1, 2, and 4). Excessive crying was calculated as crying for ≥3 h/d for ≥3 d/wk, similar to the current Rome IV clinical research criteria [3]. In addition, mean participant age at the start of study feeding (∼42 d) was consistent with the age when excessive crying, or infantile colic, also peaks [4]. Results indicate the INV-PHP formula is suitable for term infants and supports infants identified as fussy with respect to crying and excessive crying.

The physical function score significantly increased (signifying less parent/caregiver fatigue and other physical attributes) [1,17] by D7 and D25 in the INV group and by D25 in the control group, corresponding to less fussiness, crying, spit-up, and fewer night-wakings on post hoc analysis. Further investigation is needed, but significant improvements for parent HRQoL summary score by D7, family functioning summary score by D25, and overall total scale score by D7 and D25 may be associated with early improvement in crying and significantly less crying across the study period in the INV group, whereas delayed improvement in the control group may be consistent with the later significant decrease in crying by week 3.

As an indicator of tolerance, stool frequency and consistency were reported throughout the study using a similar scale to past studies [10]. In this study, stool consistency was reported between soft and unformed/seedy and stool frequency between 2 and 3.5 times per day in both groups over the study period. Such stool frequency and consistency are typical in breastfed infants and infants who receive formula supplemented with prebiotics when compared with those who receive nonsupplemented formulas [[20], [21], [22]]. The incidence of AEs was also low and not different between study groups. In a separate study, these formulas were also shown in term infants to support age-appropriate growth and be well tolerated [23]. These findings are consistent with other formulas that have a prebiotic blend of PDX:GOS, previously shown to be well tolerated, support age-appropriate growth, and promote soft stools [13,15,24,25] closer to those of infants receiving human milk [11]. Current results indicate that PHP formulas (hydrolyzed whey and casein) with PDX:GOS are appropriate first-switch formulas for infants identified as fussy, building upon previous data [9].

Whereas responses to 2 common switch formulas (PHP and soy protein-based formulas marketed prior to addition of prebiotics) were previously evaluated in very and extremely fussy infants [9], the present study included a control, intact protein formula, and expanded the study population to moderately fussy infants; both study formulations had prebiotics, and additional outcomes were assessed. Similar to the previous study [9], post hoc analysis was performed in the current study, which revealed that mean fussiness scores, gassiness, and spit-up significantly decreased within 1 d of starting study feeding within both groups. Improvements were maintained throughout the feeding period and by study end. Additionally, night-wakings significantly decreased within each group and sleep quality significantly increased within each group, still falling within age-appropriate sleep patterns [18,26]. Infant discomfort is generally believed to improve with time [27], but switching formulas, among healthy term infants who are not experiencing intolerance related to allergy or other clinical indications, is a common practice in general pediatrics. The present trial and another study [9] indicate the act of switching formulas may support rapid improvement in parent-perceived comfort relative to baseline measures and may be important to consider in future clinical trial designs.

Although the aim of this trial was to compare a PHP formula with an intact cow milk protein formula, there were some limitations. To ensure nutritional suitability, the protein content of the INV-PHP formula was increased, remaining within expert recommendations [10,23]. Based on past and recent studies supporting nutritional suitability and tolerance of the same protein hydrolysate, this difference in protein amount between the study formulas should not be expected to impact tolerance measures. Furthermore, micronutrients with potential to impact tolerance, such as iron and magnesium, were held constant between the 2 formulas, whereas other minor differences in micronutrient composition result from inherent contribution from raw materials and are within expert recommendations for the composition of formulas for infants [[28], [29], [30], [31]]. PHP formulas have lower lactose than intact protein formulas due to the probability for Maillard reactions with PHPs [32,33] and are hypothesized to decrease the load of undigested protein and lactose reaching colonic bacteria [34,35], thus lessening the amounts that could be fermented to produce gas and promote intestinal discomfort. The literature on PHP formulas does not point to a single efficacious level of lactose, and the present trial was not designed to test the effect of varying the lactose levels in PHP formulas. Although both arms in this study included PDX:GOS, precluding a comparison with non-PDX/non-GOS containing formula, this design reflects the current landscape in which most infant formulas include prebiotics. Additionally, although study outcomes were parent-reported compared with physician-reported, similar clinical outcomes have been previously reported in infants [[9], [10], [11],^13^,^24^,^25^,^36^,^37^], and validated questionnaires were used to report sleep and quality of life [17,18]. In addition, physicians use parent-reported data to aid in diagnoses, as evidenced by Rome IV criteria for infant colic [3]. Lastly, although some analyses were conducted post hoc, this approach is clearly stated in the Methods section, and all clinical trial results are available in this publication.

The present study demonstrates that INV-PHP formula with added PDX:GOS did not reduce mean fussiness but did reduce crying and excessive crying compared with control intact protein formula, among moderately to excessively fussy infants. In addition, decreased fussiness, gassiness and spit-up, and improved sleep and parent quality of life were observed within the INV-PHP and control groups, indicating safety and suitability in the studied population.

## Author contributions

The authors’ responsibilities were as follows—SSW: conceptualized the study; SSW, VF, NM, WZ, JLW: performed methodology; PB, SB, CD, SJ, NK, KM, FO, MY: investigated the study; WZ: curated the data and performed formal analysis; VF, MMBB, ACP, WZ, JLW: wrote the original draft; PB, SB, SC, CD, SJ, NK, KM, FO, MY, SSW, VF, MMBB, NM, ACP, WZ, and JLW: reviewed and edited the manuscript;, SSW, VF: supervised the study; and all authors: read and approved the final manuscript.

## Data availability

The authors and study sponsor encourage and support the responsible and ethical sharing of data from clinical trials. De-identified participant data described in the manuscript, code book, and analytic code will be made available upon a reasonable request pending a Data Use Agreement. Requests may be directed to: weihong.zhuang@reckitt.com.

## Funding

Study funding was provided by the study sponsor, Mead Johnson Nutrition.

## Conflict of interest

VF, MMBB, SC, NM, ACP, and WZ are employed by the study sponsor, Mead Johnson Nutrition. SSW and JLW were previously employed by the study sponsor. PB, SB, CD, SJ, NK, KM, FO, and MY were provided funding in order to independently enroll study participants.
