# Supplementary material for: A Randomized Controlled Trial of a Partially Hydrolyzed Formula on Comfort Measures in Fussy Infants
Source: Curr Dev Nutr. 2025 Oct 13;9(11):107574. doi: 10.1016/j.cdnut.2025.107574 (PMC12662101; doi:10.1016/j.cdnut.2025.107574)
Supplement: multimedia component 1 [file mmc1.docx]

**Supplementary Materials**

A Partially Hydrolyzed Formula on Comfort Measures in Fussy Infants: RCT

Veronica Fabrizio, et al.

Evidence Generation and Clinical Research, Mead Johnson Nutrition, Evansville, IN, USA

**Supplemental Table 1.** Daily Diary Questionnaire

| **Daily Diary Questions** | **Responses** |
| --- | --- |
| 1. From the time your baby woke up this morning to 4 PM this afternoon, how fussy has your baby been? | Not at all fussy = 0  Slightly fussy = 1  Moderately fussy = 2  Very fussy = 3  Extremely fussy = 4 |
| 2. From 4 PM this afternoon up to the time you put your baby to bed for the night, how fussy has your baby been? | Not at all fussy = 0  Slightly fussy = 1  Moderately fussy = 2  Very fussy = 3  Extremely fussy = 4 |
| 3. Over the past 24 hours, approximately how many hours did your baby cry? | Hours/Day |
| 4. Over the past 24 hours, how many times did your baby spit-up? | Number/Day |
| 5. How many bowel movements did your baby have in the past 24 hours? | Number/Day |
| 6. What category best represents the consistency of your baby’s stool over the past 24 hours? (Use pictures provided for reference.) | Hard = 1  Formed = 2  Soft = 3  Unformed or seedy = 4  Watery = 5 |
| 7. Over the past 24 hours, how much gas has your baby had? | None = 1  Slight = 2  Moderate = 3  Excessive = 4 |
| 8. How many times did your baby wake last night? | Number/Night |
| 9. How well did your baby sleep last night? | Very well = 0  Well=1  Fairly well=2  Poorly=3  Very poorly=4 |
| 10. Over the past 24 hours, did you give your baby any nonprescription products to help relieve pain, fussiness, spitting up, constipation, or gassiness? | If Yes, what did you give? |

**Supplemental Table 2.** Pre-defined and post-hoc outcome analyses.

|  | **Outcome Measures** | **Pre-defined Analysis,**  **between groups** | **Post-hoc Analysis,**  **within groups** |
| --- | --- | --- | --- |
| **Primary Outcome** | | | |
| Fussiness | Fussiness scores at study feeding day 1 through 7 | X | X |
| **Secondary Outcomes** | | | |
| Fussiness | Fussiness scores at study feeding weeks 1, 2, 3, and 4 | X | X |
| Gassiness | Gassiness scores during the first week of study feeding | X | X |
|  | Gassiness scores at study feeding weeks 1, 2, 3, and 4 | X | X |
| Spit-up | Number of spit-up per day during the first week of study feeding between groups | X | X |
|  | Number of spit-up per day at study feeding weeks 1, 2, 3, and 4 | X | X |
| Crying | Hours of crying per day during the first week of study feeding | X | X |
|  | Hours of crying at study feeding weeks 1, 2, 3, and 4 | X | X |
|  | Participants who cried 3 or more hours per day for 3 or more days during each study feeding week | X |  |
| Stool frequency | Stool frequency during the first week of study feeding | X | X |
|  | Stool frequency at study feeding weeks 1, 2, 3, and 4 | X | X |
| Stool consistency | Stool consistency scores during the first week of study feeding | X | X |
|  | Stool consistency scores at study feeding weeks 1, 2, 3, and 4 | X | X |
| Night wakings | Number of night wakings during the first week of study feeding | X | X |
|  | Number of night wakings at study feeding weeks 1, 2, 3, and 4 | X | X |
| Nighttime sleep quality | Nighttime sleep quality scores during the first week of study feeding | X | X |
|  | Nighttime sleep quality scores at study feeding weeks 1, 2, 3, and 4 between groups | X | X |
| Brief Infant Sleep Questionnaire | BISQ-R SF scores at study feeding days 14 and 25 | X | X |
| Pediatric Quality of Life | PedsQL FIM - acute scores at study feeding days 7 and 25 | X | X |
| Study formula intake | Study Formula Intake (oz/day) at Study Feeding Days 7 and 25 | X |  |
| Medically confirmed adverse event | Number of participants for whom at least one adverse events was reported | X |  |
|  | Adverse events by body system and event | X |  |

BISQ-R SF: Brief Infant Sleep Questionnaire-Revised Short Form; PedsQL FIM: Pediatric Quality of Life Inventory™ Family Impact Module

**Supplemental Table 3A**. Number of participants responded on daily diary questionnaire for the first 7 study days

| Outcome |  | D0 | D1 | D2 | D3 | D4 | D5 | D6 | D7 |
| --- | --- | --- | --- | --- | --- | --- | --- | --- | --- |
| Fussiness | Control | 70 | 60 | 55 | 65 | 61 | 59 | 59 | 55 |
|  | INV-PHP | 72 | 59 | 50 | 61 | 51 | 50 | 49 | 49 |

Control: Intact protein formula; D: Day(s); INV-PHP: investigational partially hydrolyzed cow’s milk protein formula

**Supplemental Table 3B**. Number of participants responded on daily diary questionnaire for each study week

| Outcome |  | Wk1 | Wk2 | Wk3 | Wk4 |
| --- | --- | --- | --- | --- | --- |
| Fussiness | Control | 71 | 66 | 62 | 64 |
|  | INV-PHP | 72 | 67 | 65 | 63 |

Control: Intact protein formula; INV-PHP: investigational partially hydrolyzed cow’s milk protein formula; Wk: Week(s)

**Supplemental Figure 1.** Mean daily values (D1 to D7) and weekly averages (Wk1 to Wk4) compared between groups and within group to D0 (baseline) for infant gassiness (1-4 scale; A and B), and spit-up (number/day; C and D), and crying (hours/day; E and F) within study groups. Control (dark blue circles) and INV-PHP (light blue squares); No between group differences were detected; * P < 0.05 for change within group from baseline value. Bar indicates standard error (SE) for means. For each of the secondary outcomes, there was a minimal variation (±4%) of the sample sizes for each study group at each time point compared to the corresponding time points shown in Supplemental Table 3 for fussiness. Day(s); Wk: Week(s); Control: Intact protein formula; INV-PHP: investigational partially hydrolyzed cow’s milk protein formula

**Supplemental Figure 2.** Mean daily values (D1 to D7) and weekly averages (Wk1 to Wk4) compared between groups and within group to D0 (baseline) for stool frequency (number/day; A and B), stool consistency (1-5 scale; C and D), night-wakings (number/night; E and F), and nighttime sleep quality (0-4; G and H) within study groups Control (dark blue circles) and INV-PHP (light blue squares); No between group differences were detected; * P < 0.05 for change within group from baseline value. Bar indicates standard error (SE) for means. For each of the secondary outcomes, there was a minimal variation (±4%) of the sample sizes for each study group at each time point compared to the corresponding time points shown in Supplemental Table 3 for fussiness. Day(s); Wk: Week(s); Control: Intact protein formula; INV-PHP: investigational partially hydrolyzed cow’s milk protein formula
